# Supplementary material for: Preparation of Methacrylate-Based Polymers Modified with Chiral Resorcinarenes and Their Evaluation as Sorbents in Norepinephrine Microextraction
Source: Polymers (Basel). 2019 Aug 30;11(9):1428. doi: 10.3390/polym11091428 (PMC6780700; doi:10.3390/polym11091428)

Supplementary Material: **Preparation of methacrylate-based polymers modified with chiral resorcinarenes and their evaluation as sorbents in norepinephrine microextraction**

Alver Castillo-Aguirre and Mauricio Maldonado

Table of Contents:

1. Preparation of Polymers

Figure S1. Chemical characterization of **1**

Figure S2. Thermal stability and morphological characterization of **1**

Figure S3. Chemical characterization of **2**

Figure S4. Thermal stability and morphological characterization of **2**

2. Synthesis of chiral resorcinarenes

Figure S5. Structure of compound **5**

Figure S6. FT-IR spectrum of compound **5**

Figure S7. ^{1}H-NMR spectrum (400 MHz, $CDCl_3$, 293 K) of compound **5**

Figure S8. ^{13}C-NMR spectrum (400 MHz, $CDCl_3$, 293 K) of compound **5**

Figure S9. DEPT 45 spectrum (400 MHz, $CDCl_3$, 293 K) of compound **5**

Figure S10. DEPT 90 spectrum (400 MHz, $CDCl_3$, 293 K) of compound **5**

Figure S11. DEPT 135 spectrum (400 MHz, $CDCl_3$, 293 K) of compound **5**

Figure S12. ^{1}H-^{1}H COSY spectrum (400 MHz, $CDCl_3$, 293 K) of compound **5**

Figure S13. HMQC spectrum (400 MHz, $CDCl_3$, 293 K) of compound **5**

Figure S14. HMBC spectrum (400 MHz, $CDCl_3$, 293 K) of compound **5**

Figure S15. Structure of compound **6**

Figure S16. FT-IR spectrum of compound **6**

Figure S17. ^{1}H-NMR spectrum (400 MHz, DMSO-d_6, 323 K) of compound **6**

Figure S18. ^{13}C-NMR spectrum (400 MHz, DMSO-d_6, 323 K) of compound **6**

Figure S19. HMQC spectrum (400 MHz, DMSO-d_6, 323 K) of compound **6**

Figure S20. RP-HPLC-UV of compound **6**

3. Polymeric Modifications of 1 and 2 with Chiral Resorcinarenes 5–7

Figure S21. Chemical characterization of **8**

Figure S22. Thermal stability and morphological characterization of **8**

Figure S23. Chemical characterization of **9**

Figure S24. Thermal stability and morphological characterization of **9**

Figure S25. Chemical characterization of **10**

Figure S26. Thermal stability and morphological characterization of **10**

4. Microextraction and Quantification of Norepinephrine

Figure S27. Screening design matrix

Figure S28. Optimization design matrix

Figure S29. Standard calibration curves. In water (0.05% TFA) (red line) and on matrix (blue line)

Figure S30. Calibration curve of fortified extracts

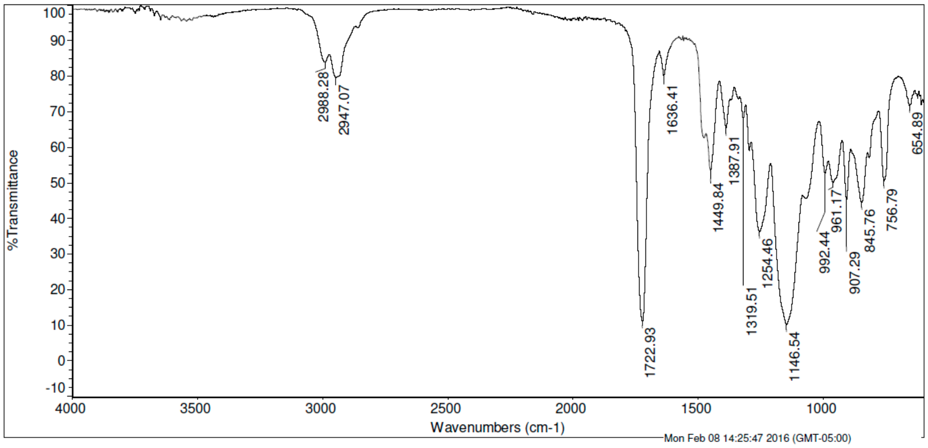

a

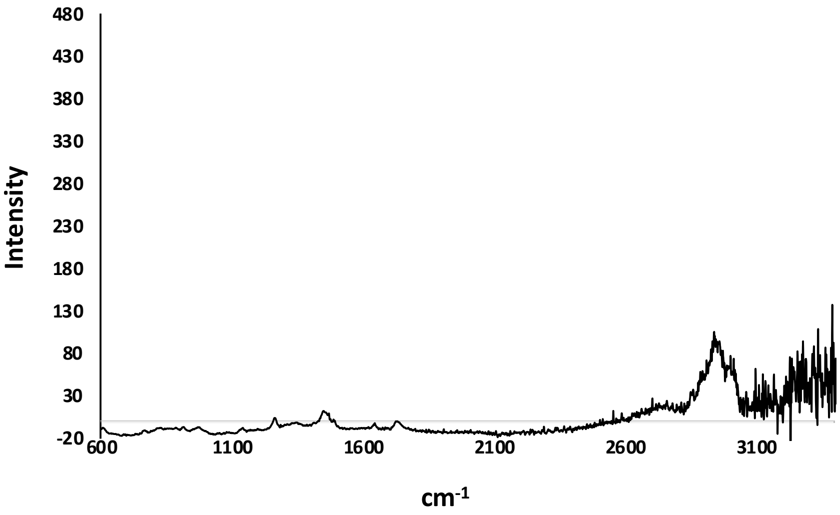

b

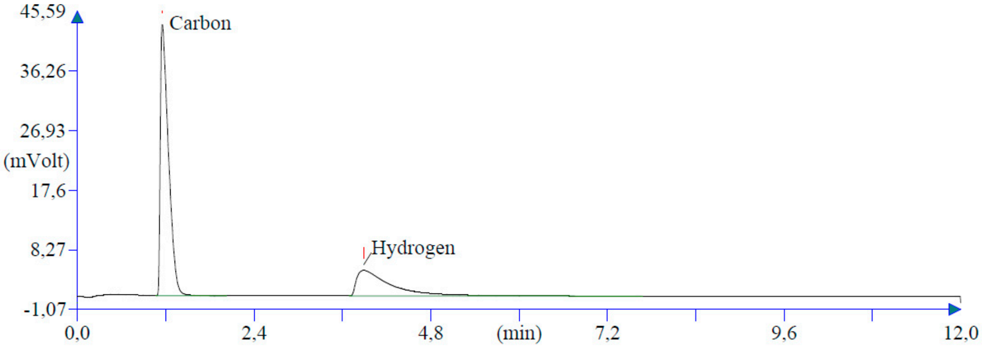

Element Name		Ret.Time	Area	BC	Area ratio	K fa
Carbon	62.0492	69	3261609	RS	1.000000	.4756
Hydrogen	7.7455	234	1299288	RS	2.510305	.1514
Totals	69.7947		4560897			

c

Figure S1. Chemical characterization of poly(GMA-*co*-EDMA) (**1**) (a) ATR-FT-IR spectra. (b) Raman spectra. (c) Elemental analysis.

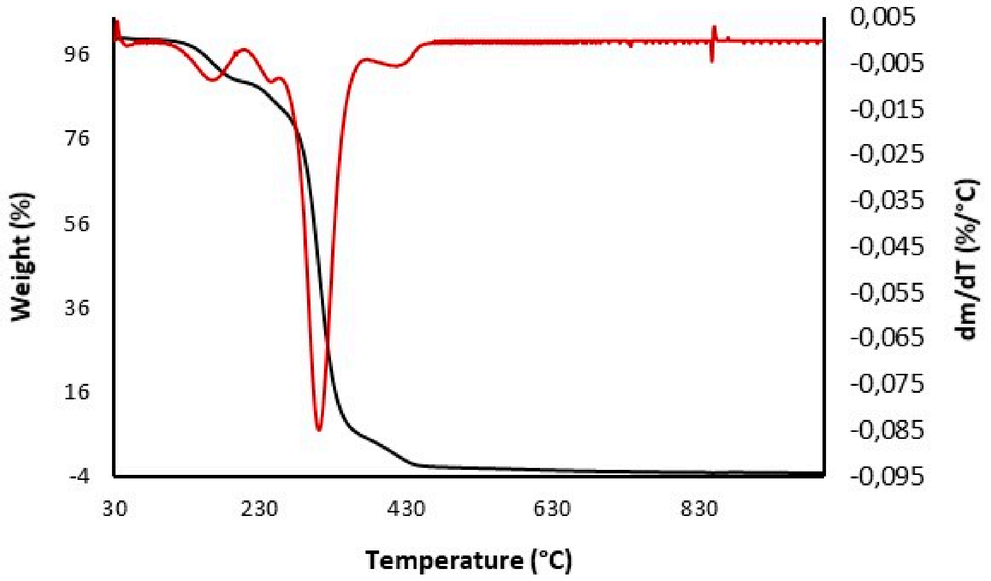

a

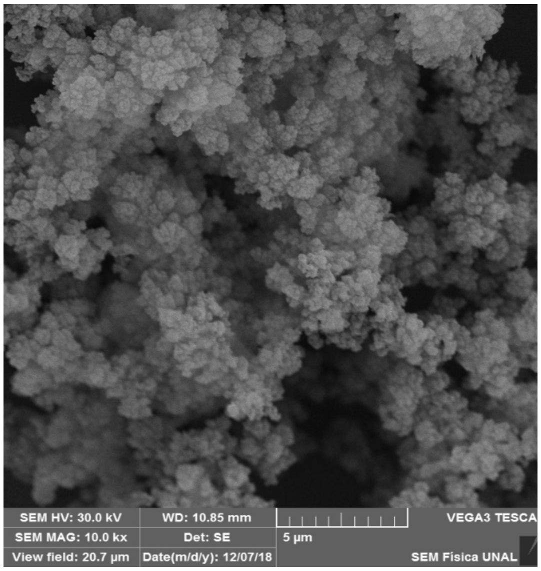

b

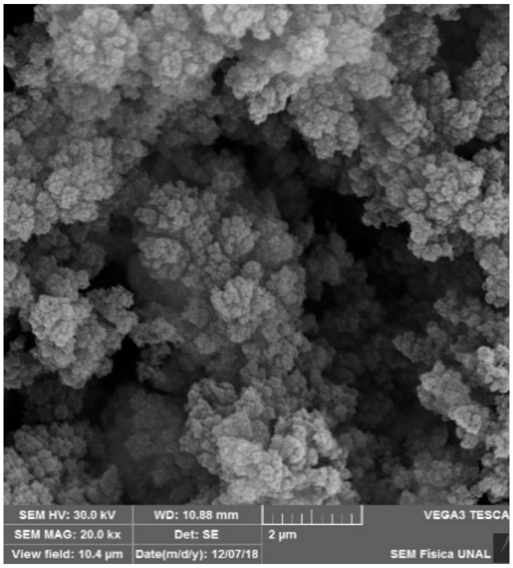

c

Figure S2. Thermal stability and morphological characterization of poly(GMA-*co*-EDMA) (**1**). (a) Thermogram TGA (black) and curve dm/dT (red). Scanning electron micrograph at (b) 5 µm and (c) 2 µm.

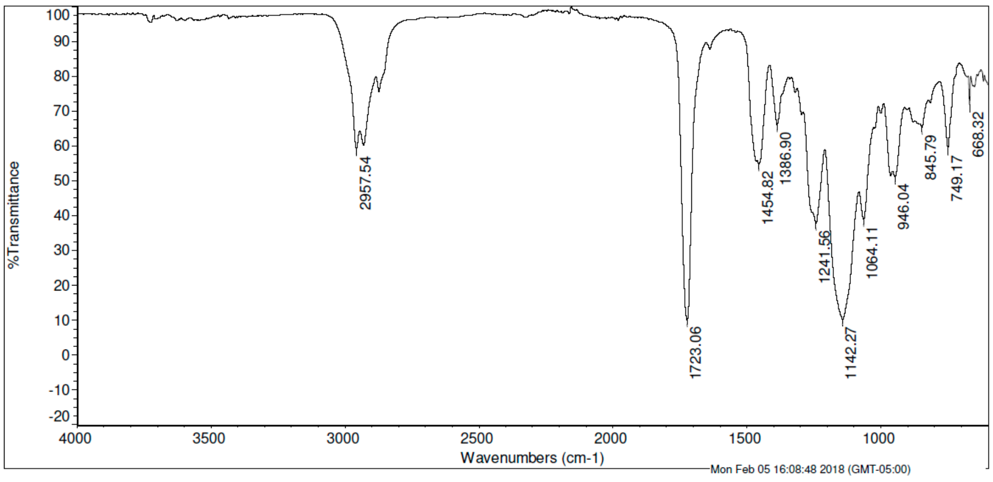

a

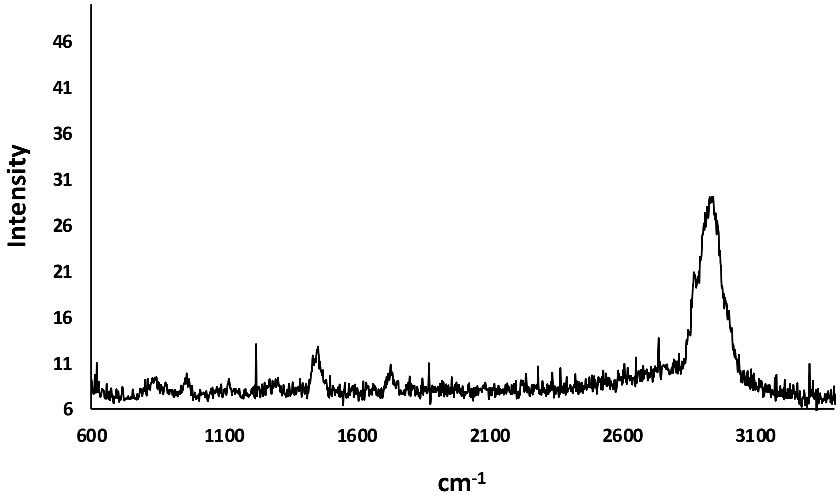

b

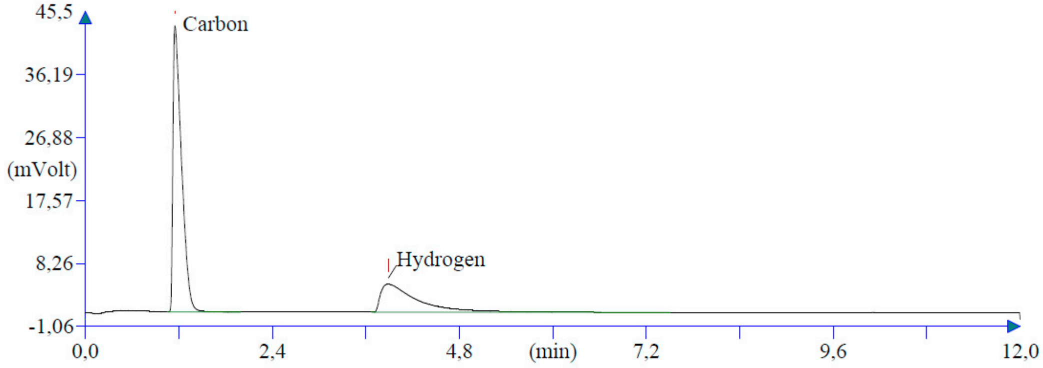

Element Name		Ret.Time	Area	BC	Area ratio	K fa
Carbon	64.4598	69	3234546	RS	1.000000	.4756
Hydrogen	8.4978	234	1360363	RS	2.377708	.1514
Totals	72.9575		4594909			

c

Figure S3. Chemical characterization of poly(BuMA-*co*-EDMA) (**2**) (a) ATR-FT-IR spectra. (b) Raman spectra. (c) Elemental analysis.

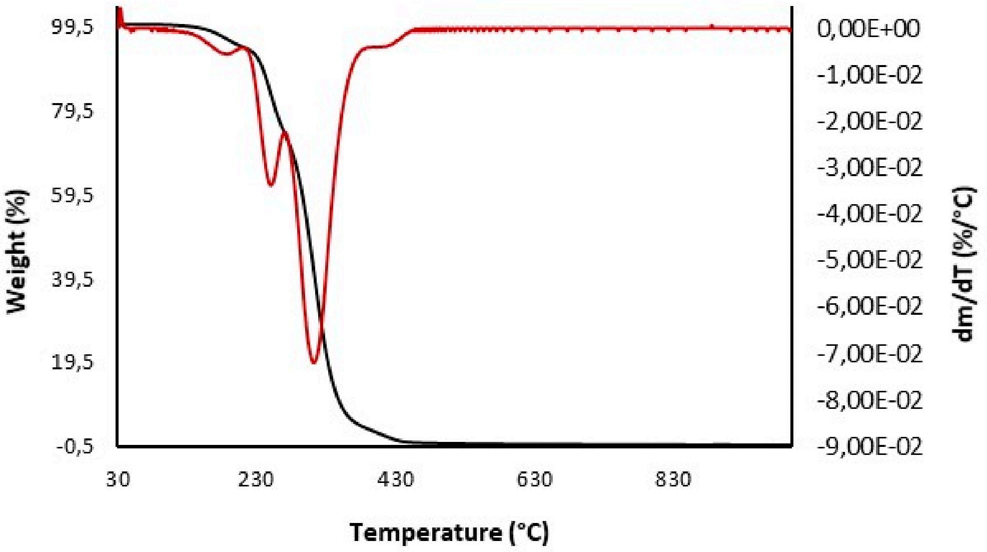

a

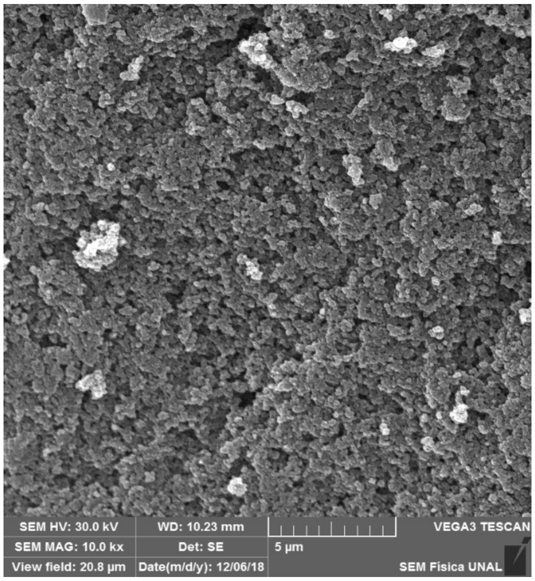

b

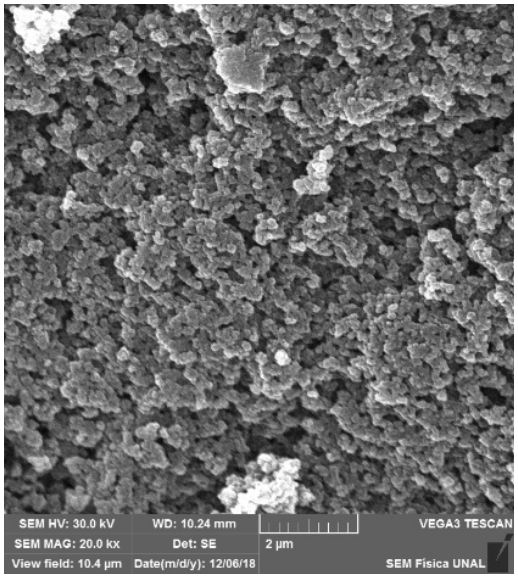

c

Figure S4. Thermal stability and morphological characterization of poly(BuMA-*co*-EDMA) (**2**). (a) Thermogram TGA (black) and curve dm/dT (red). Scanning electron micrograph at (b) 5 µm and (c) 2 µm.

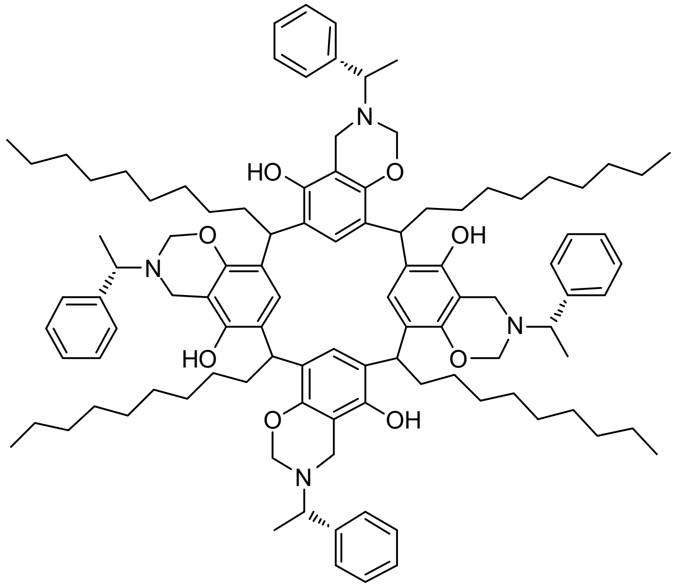

Figure S5. Structure of compound **5.**

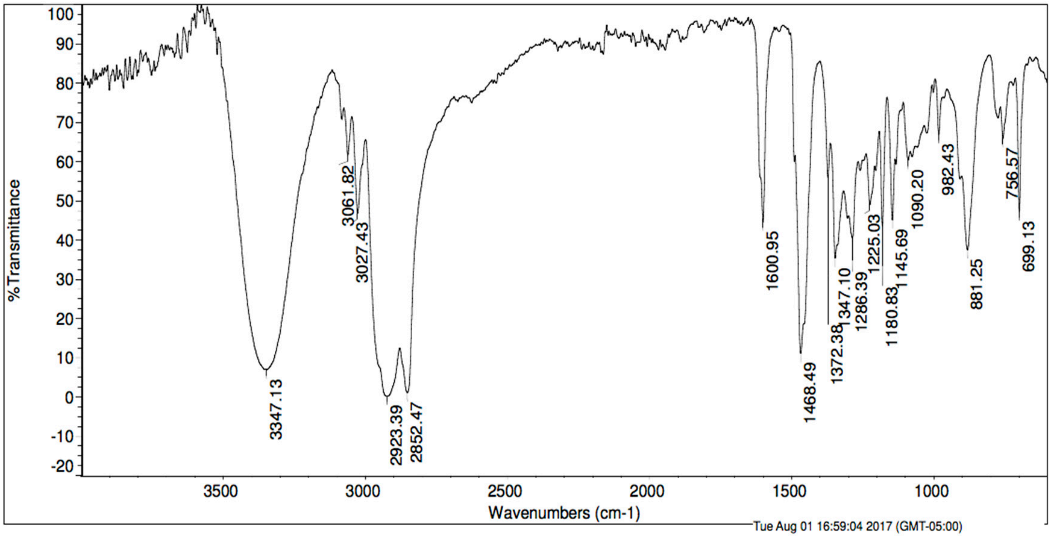

Figure S6. FT-IR spectrum of compound **5**.

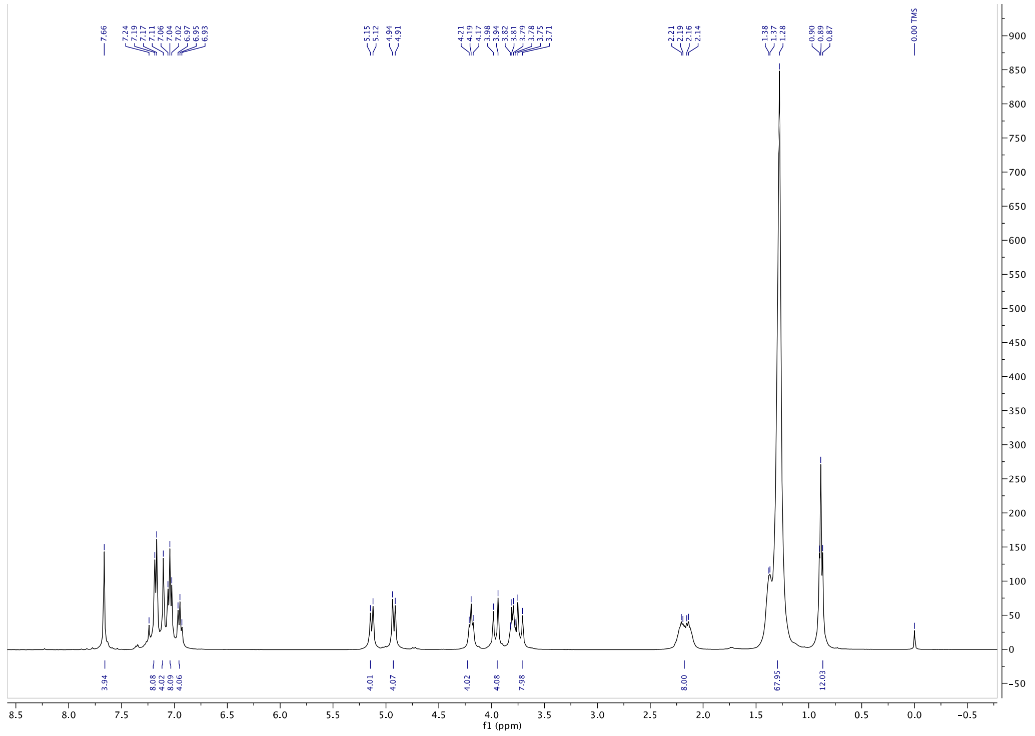

Figure S7. ^{1}H-NMR spectrum (400 MHz, $CDCl_3$, 293 K) of compound **5**.

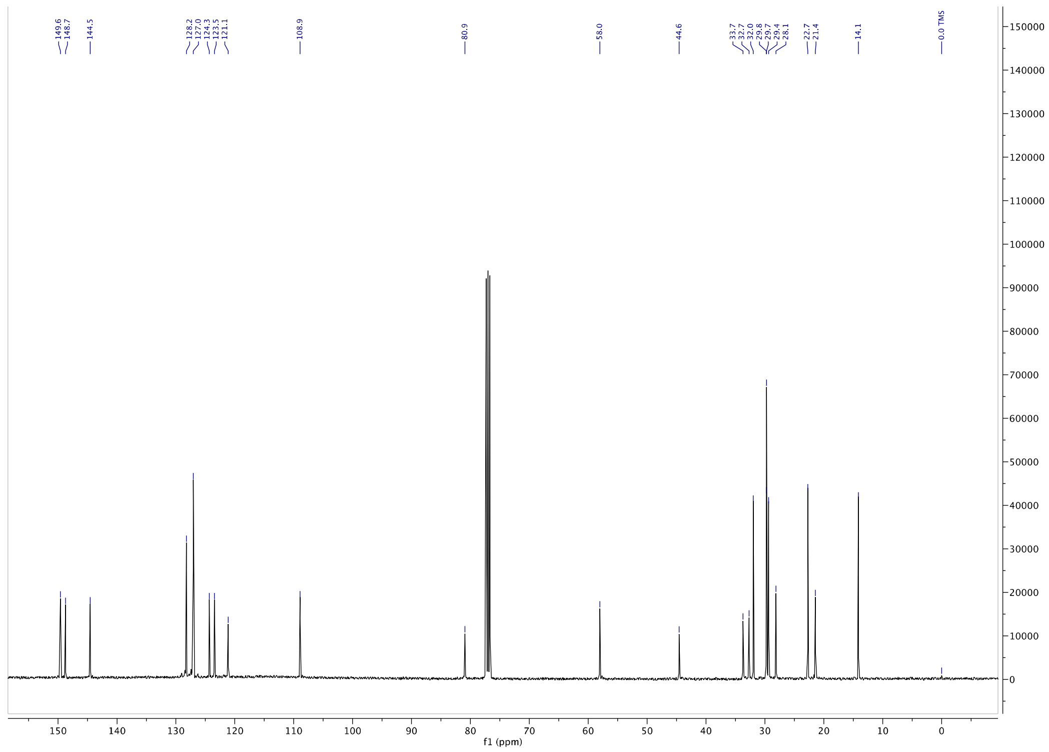

Figure S8. ^{13}C-NMR spectrum (400 MHz, $CDCl_3$, 293 K) of compound **5**.

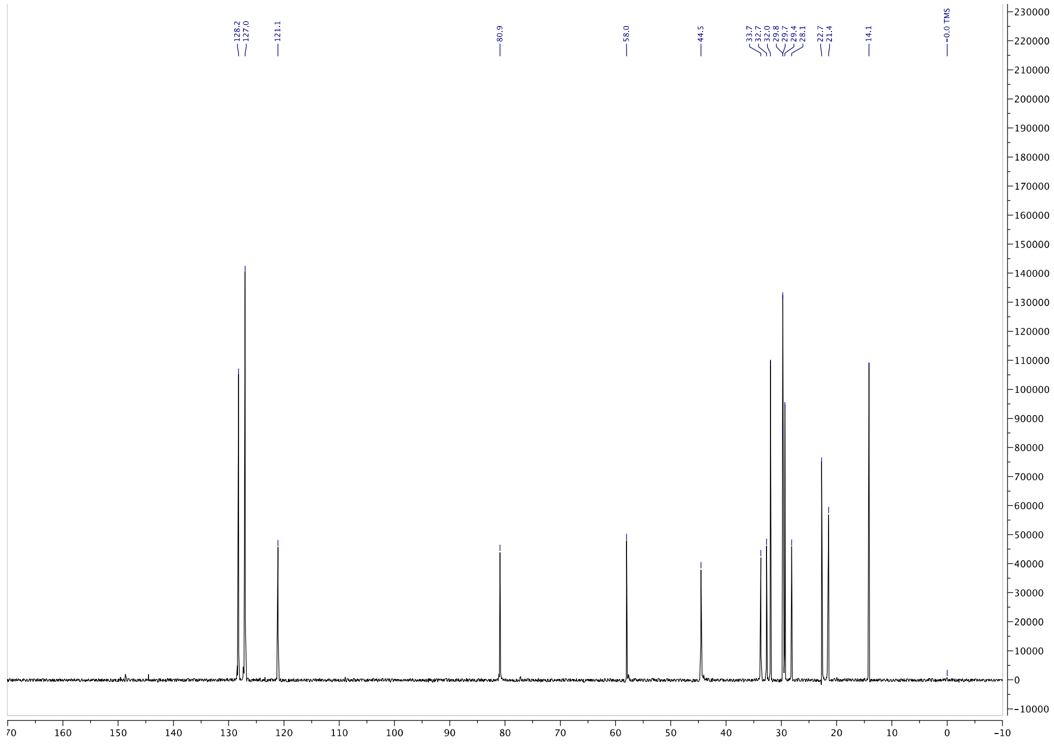

Figure S9. DEPT 45 spectrum (400 MHz, $CDCl_3$, 293 K) of compound **5**.

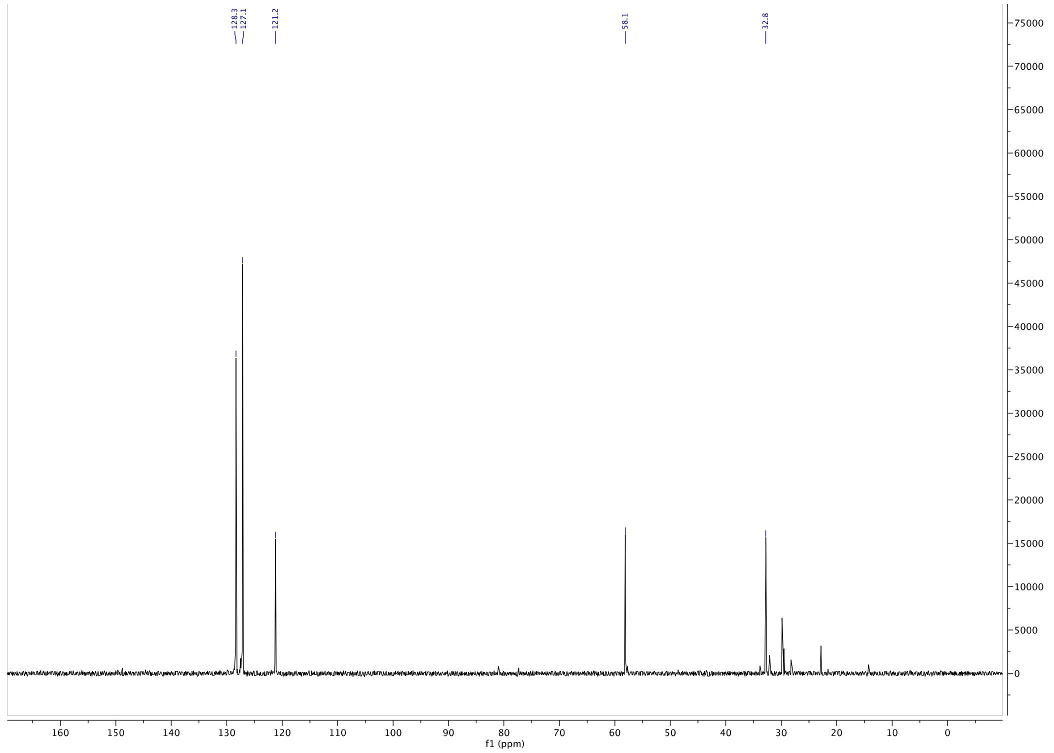

Figure S10. DEPT 90 spectrum (400 MHz, $CDCl_3$, 293 K) of compound **5**.

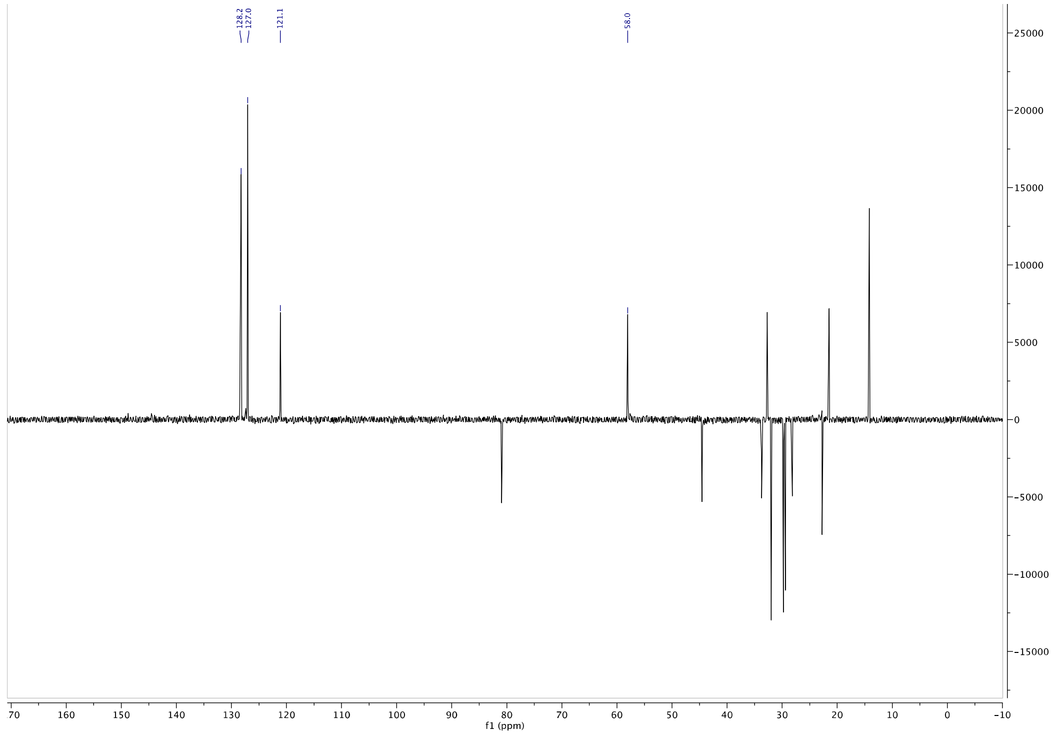

Figure S11. DEPT 135 spectrum (400 MHz, $CDCl_3$, 293 K) of compound **5**.

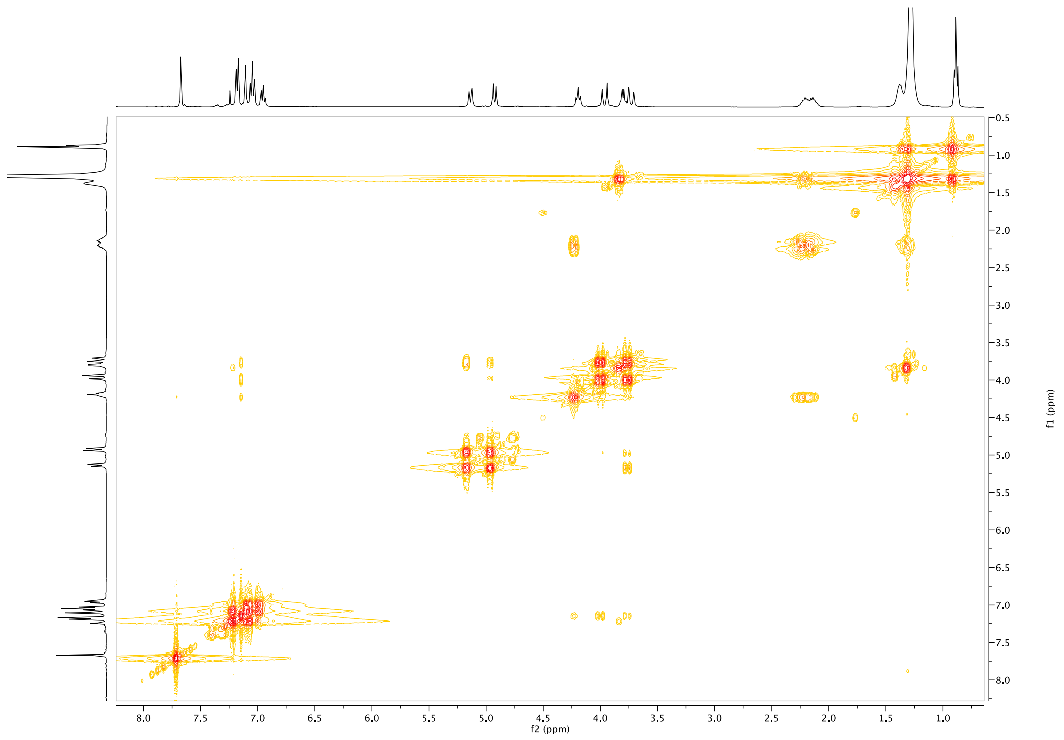

Figure S12. ^1H-^1H COSY NMR spectrum (400 MHz, $CDCl_3$, 293 K) of compound **5**.

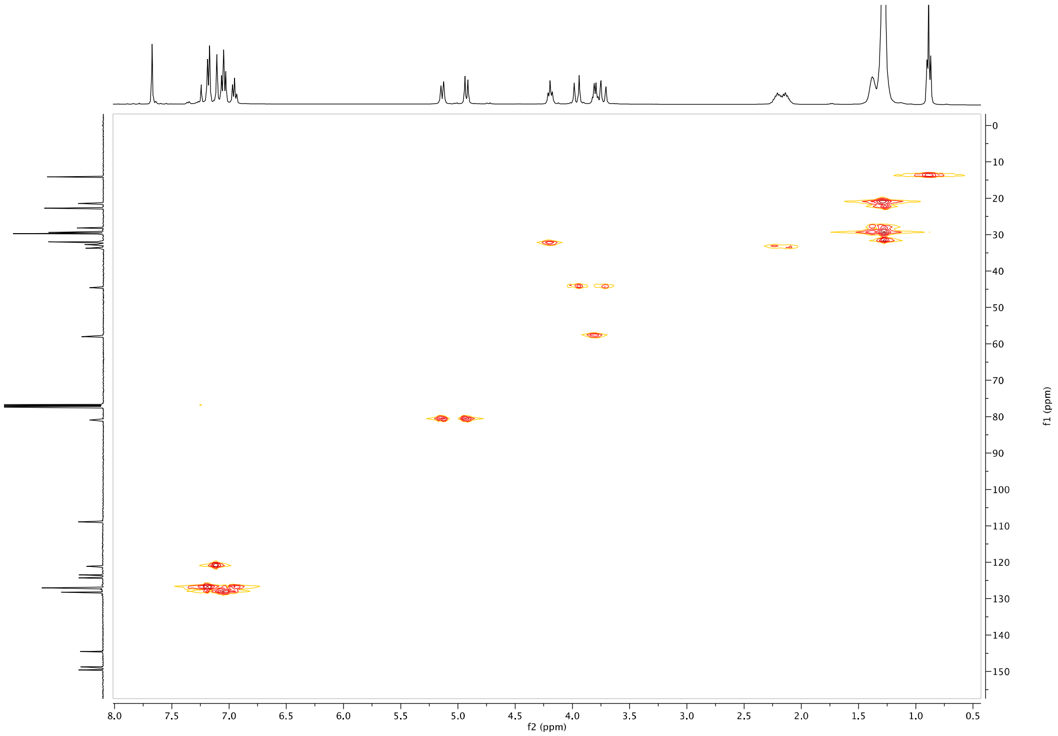

Figure S13. HMQC NMR spectrum (400 MHz, $CDCl_3$, 293 K) of compound **5**.

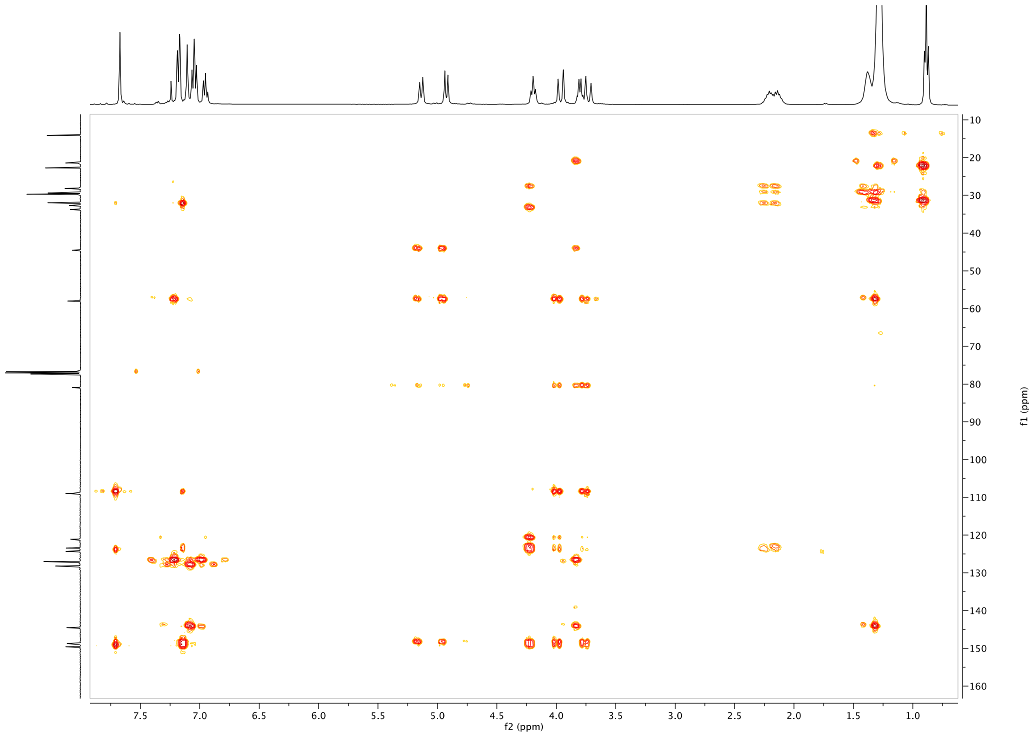

Figure S14. HMBC NMR spectrum (400 MHz, $CDCl_3$, 293 K) of compound **5**.

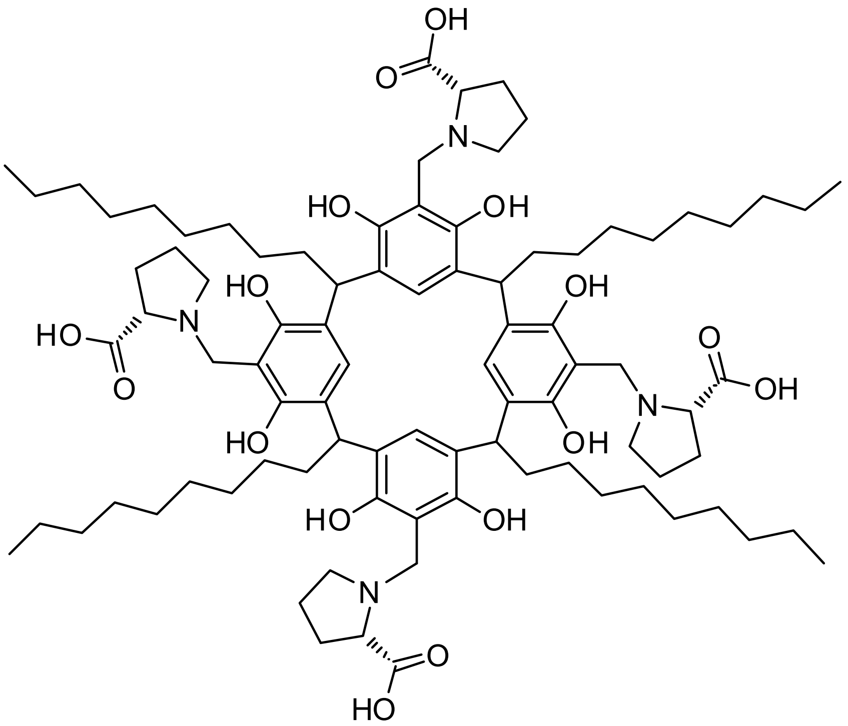

Figure S15. Structure of compound **6.**

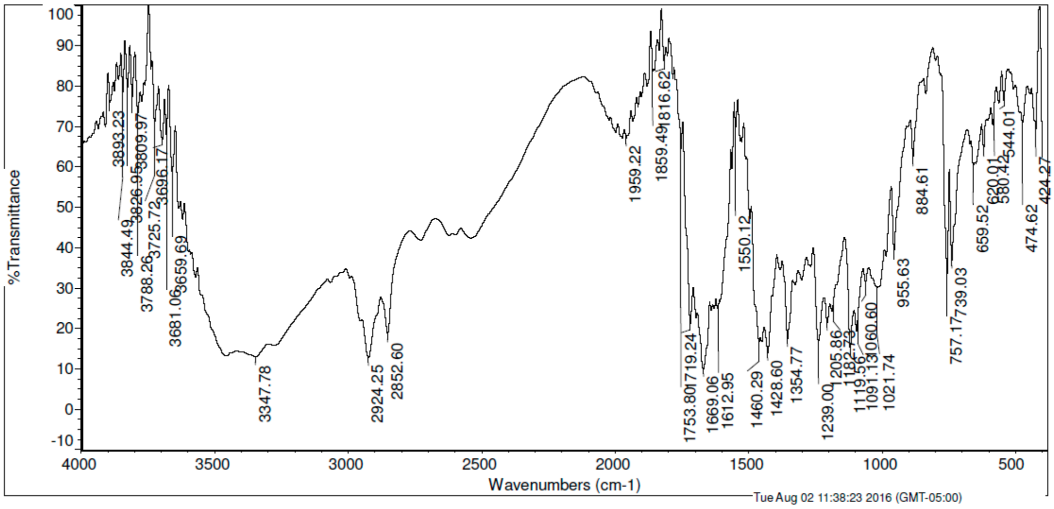

Figure S16. FT-IR spectrum of compound **6**.

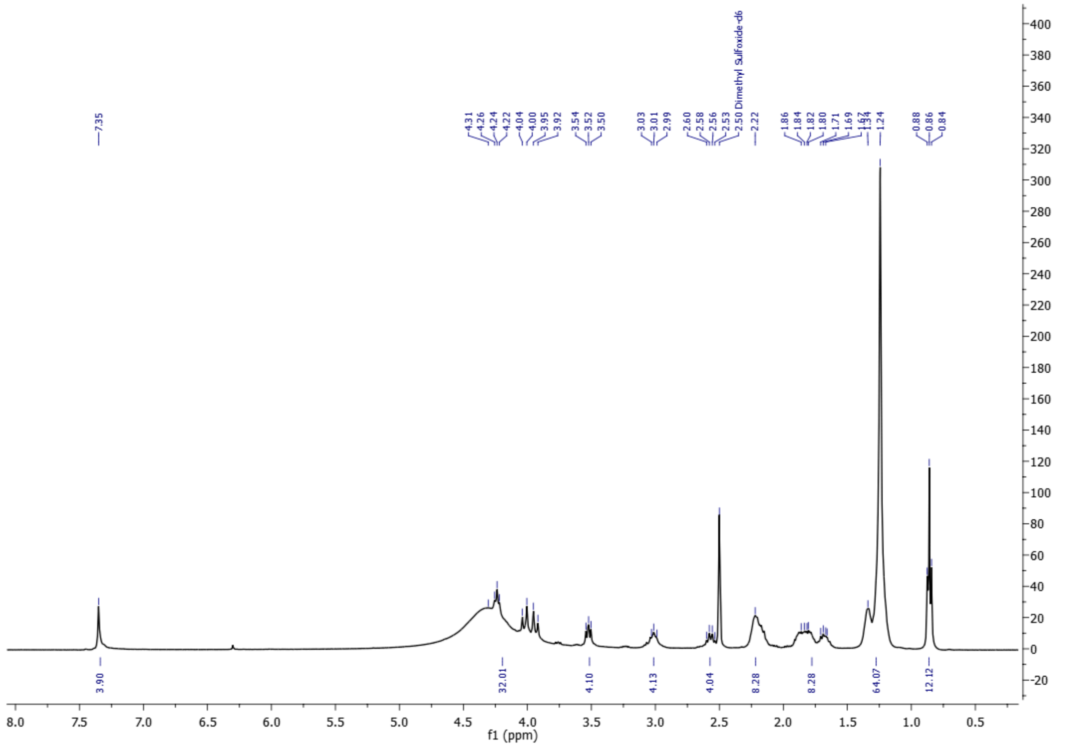

Figure S17. ^{1}H-NMR spectrum (400 MHz, DMSO-*d*6, 323 K) of compound **6**.

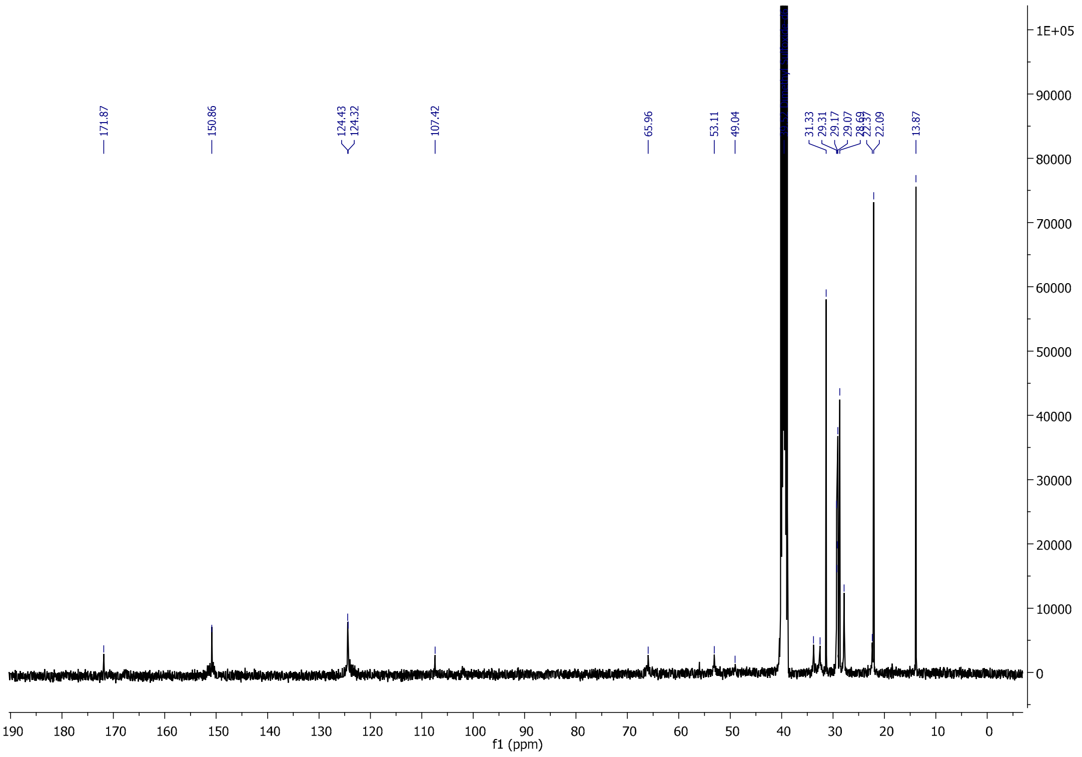

Figure S18. ^{13}C-NMR spectrum (400 MHz, DMSO-*d*6, 323 K) of compound **6**.

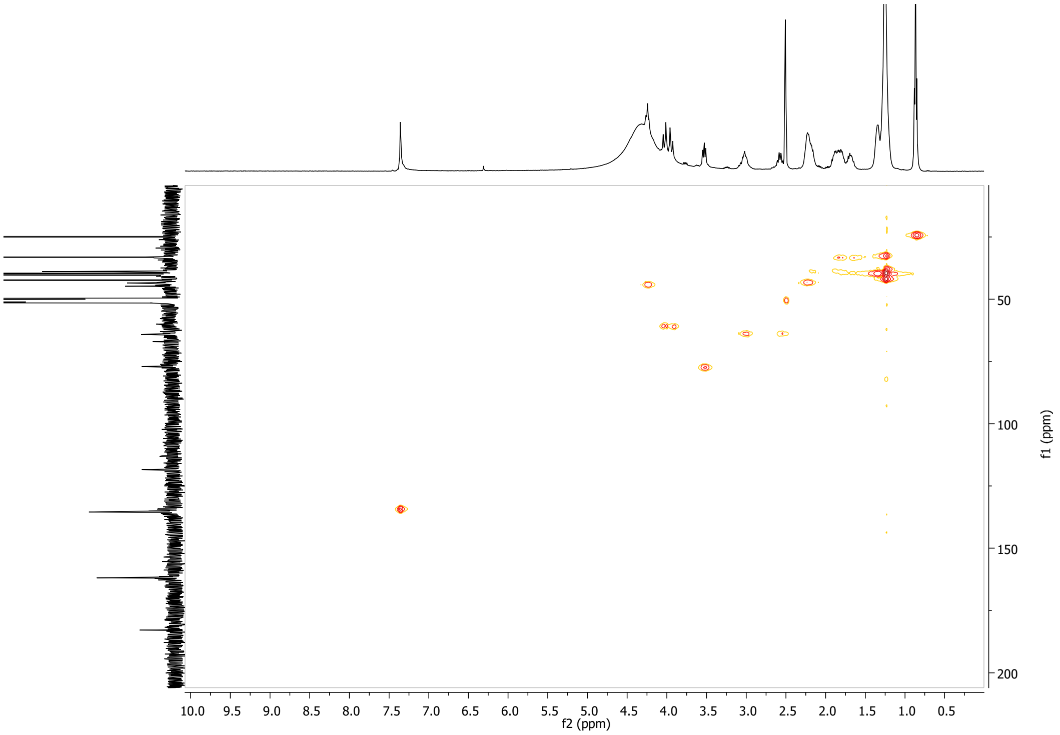

Figure S19. HMQC spectrum (400 MHz, DMSO-d_6, 323 K) of compound **6**.

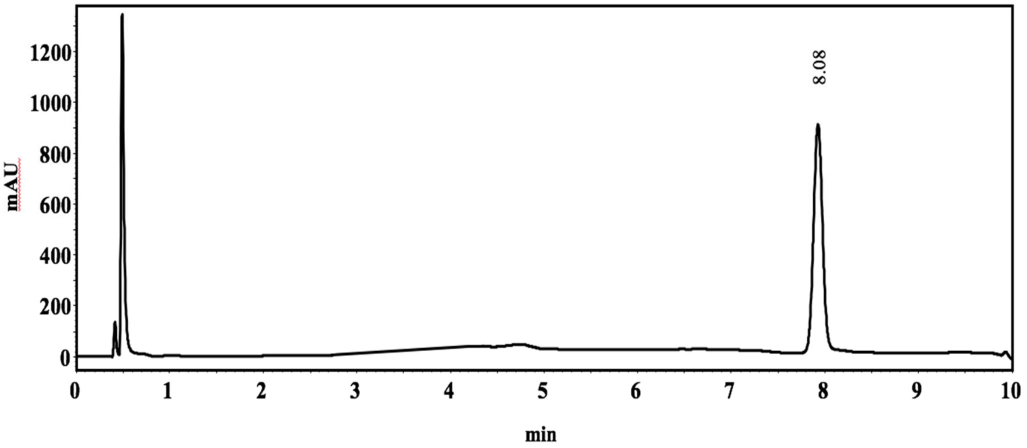

Figure S20. RP-HPLC-UV of compound **6**.

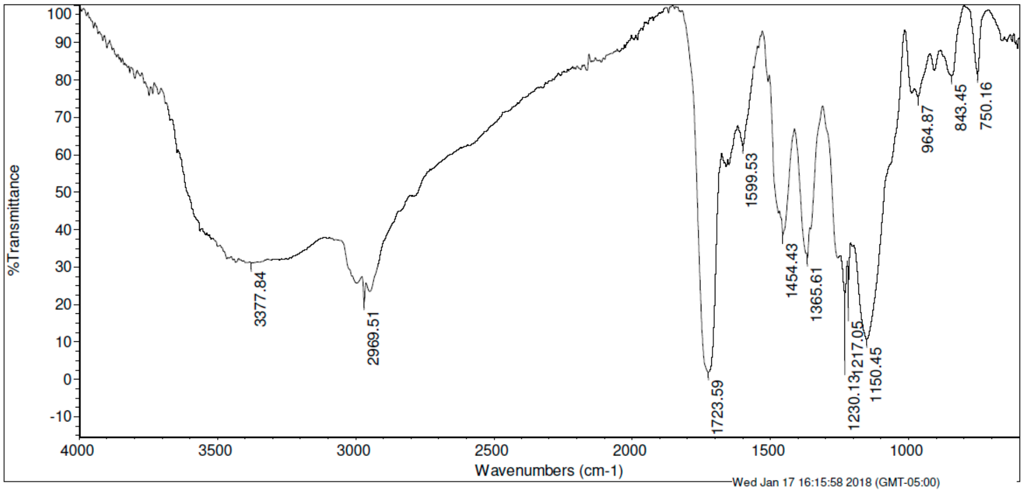

a

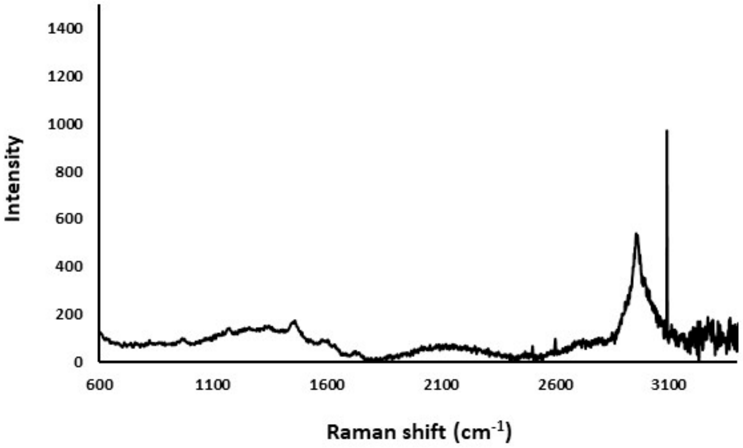

b

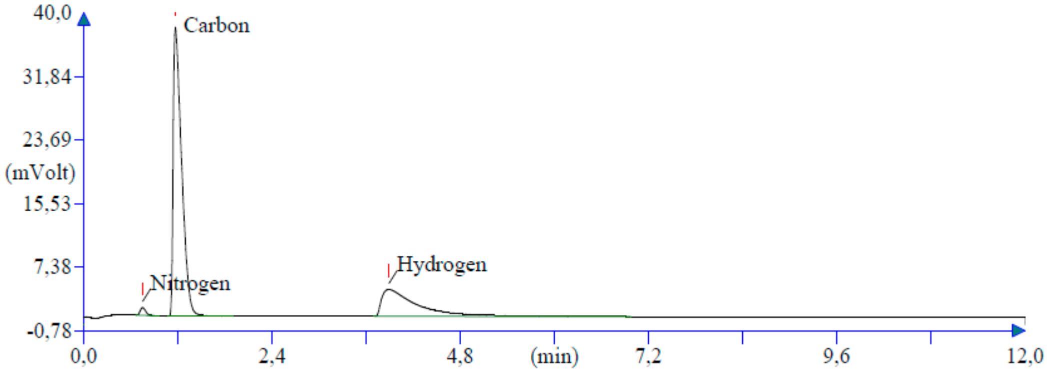

Element Name		Ret.Time	Area	BC	Area ratio	K fa
Nitrogen	2.1930	45	46276	RS	59.975910	.2367
Carbon	53.2984	70	2775445	RS	1.000000	.4756
Hydrogen	6.5224	234	1084478	RS	2.559245	.1514
Totals	61.6138		3906199			

c

Figure S21. Chemical characterization of **7**-poly(GMA-*co*-EDMA) (**8**). (a) ATR-FT-IR spectra. (b) Raman spectra. (c) Elemental analysis.

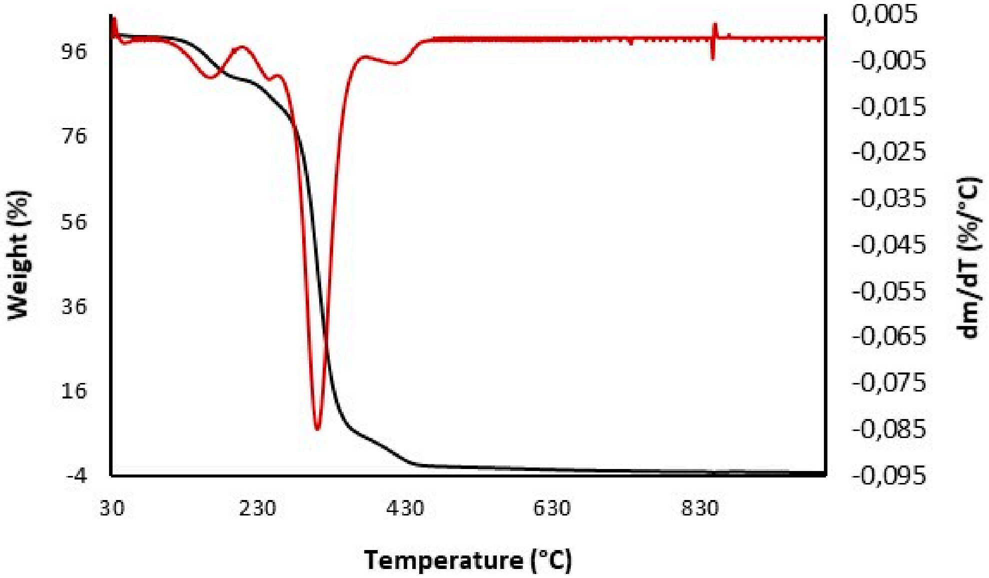

a

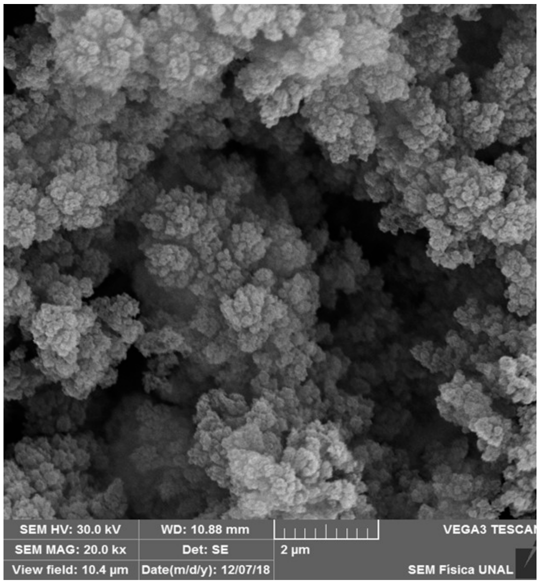

b

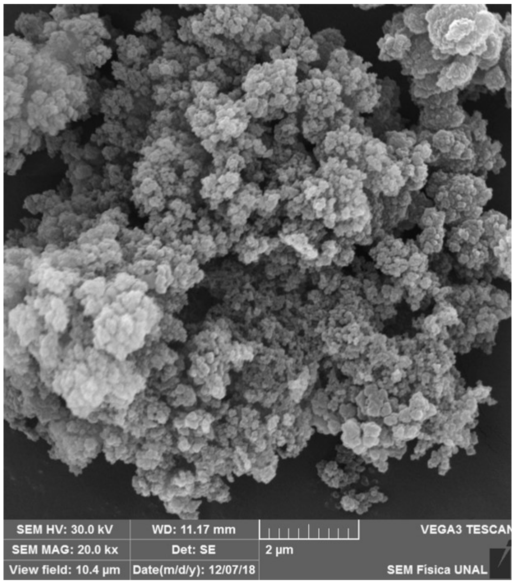

c

Figure S22. Thermal stability and morphological characterization of **7**-poly(GMA-*co*-EDMA) (**8**). (a) Thermogram TGA (black) and curve dm/dT (red). Scanning electron micrograph at (b) 5 µm and (c) 2 µm.

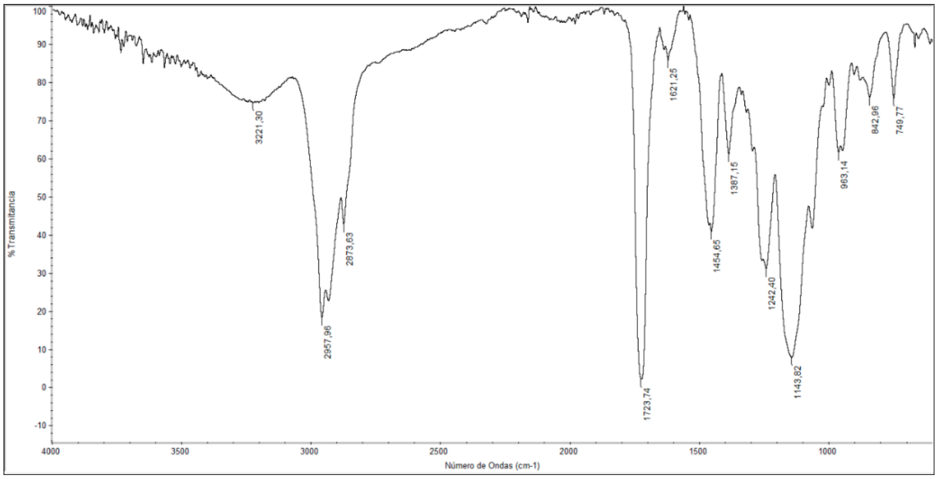

a

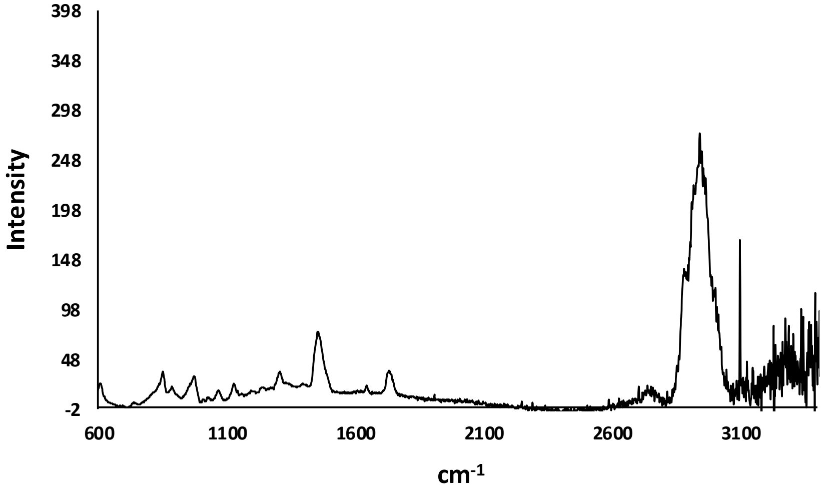

b

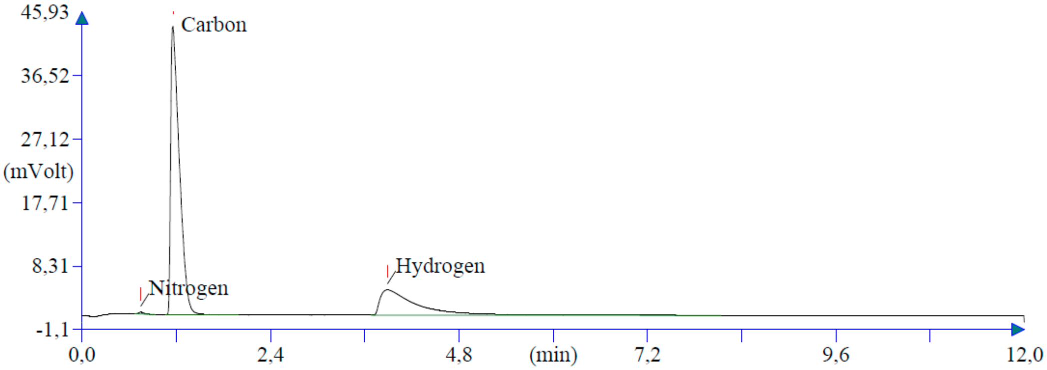

Element Name		Ret.Time	Area	BC	Area ratio	K fa
Nitrogen	0.5445	45	12577	RS	260.963200	.2367
Carbon	57.5294	70	3282134	RS	1.000000	.4756
Hydrogen	6.5997	234	1202191	RS	2.730127	.1514
Totals	64.5737		4496902			

c

Figure S23. Chemical characterization of **5**-poly(BuMA-*co*-EDMA) (**9**). (a) ATR-FT-IR spectra. (b) Raman spectra. (c) Elemental analysis.

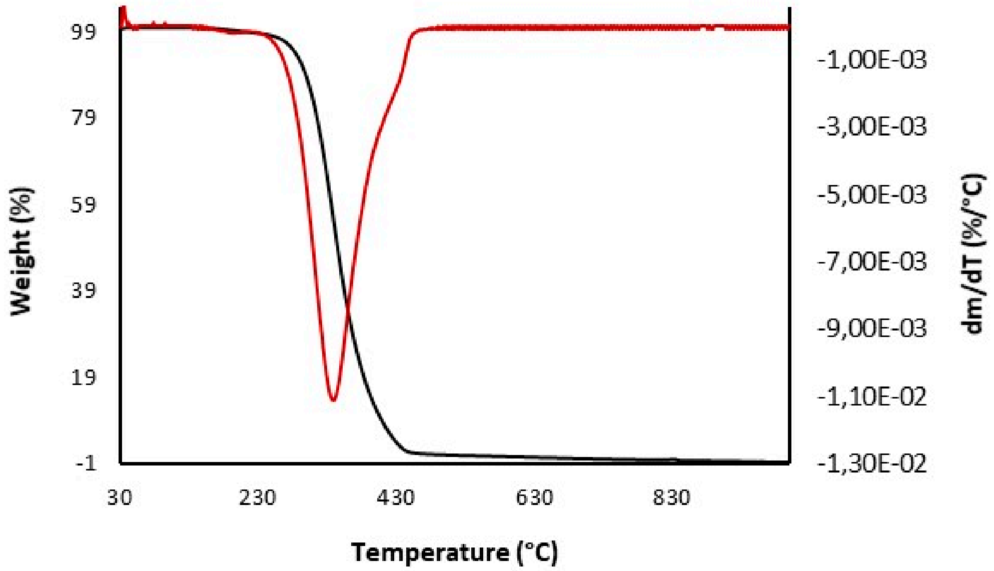

a

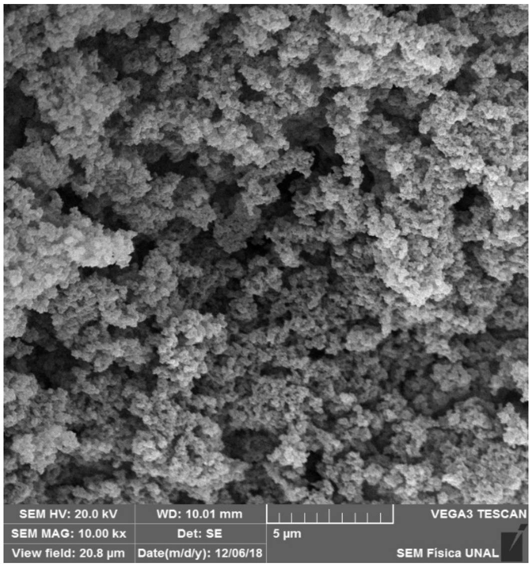

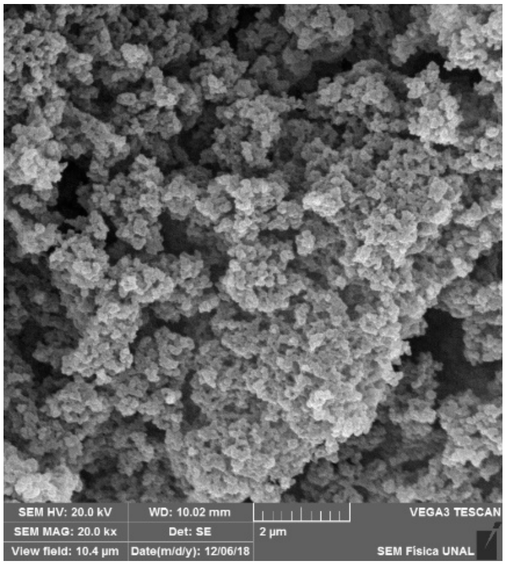

b c

Figure S24. Thermal stability and morphological characterization of **5**-poly(BuMA-*co*-EDMA) (**9**). (a) Thermogram TGA (black) and curve dm/dT (red). Scanning electron micrograph at (b) 5 μm and (c) 2 μm.

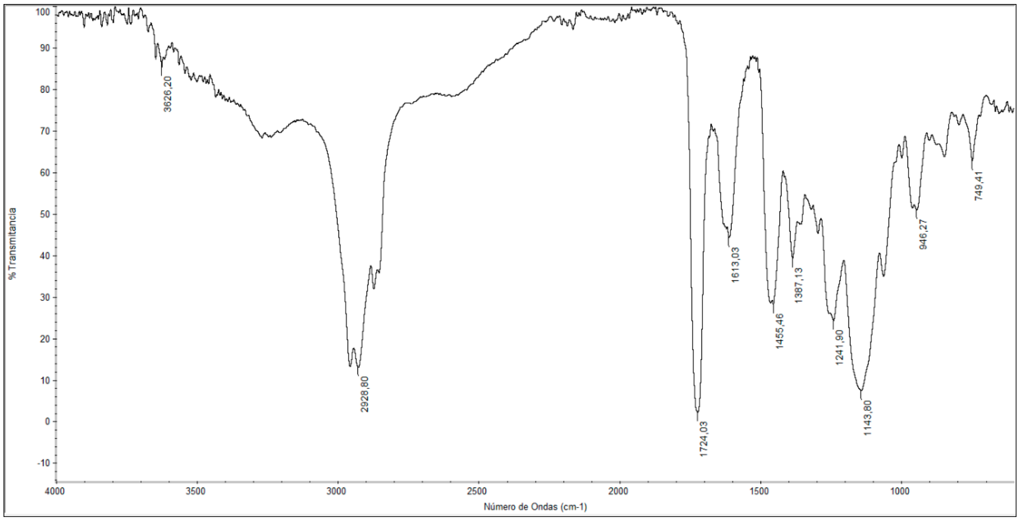

a

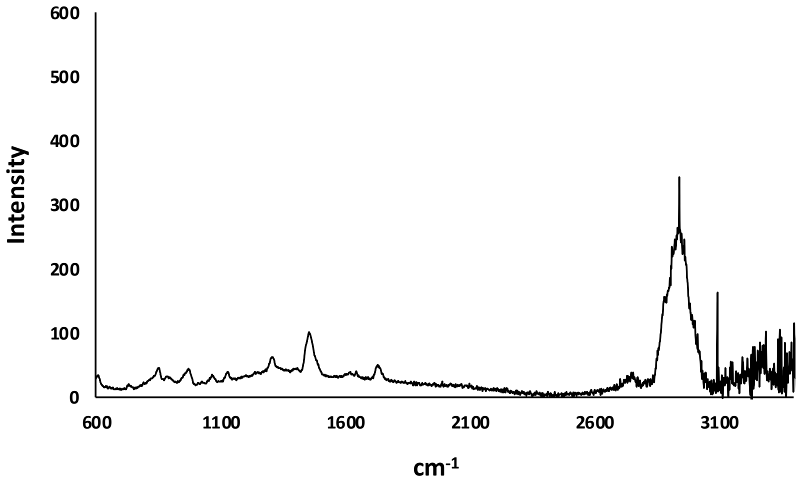

b

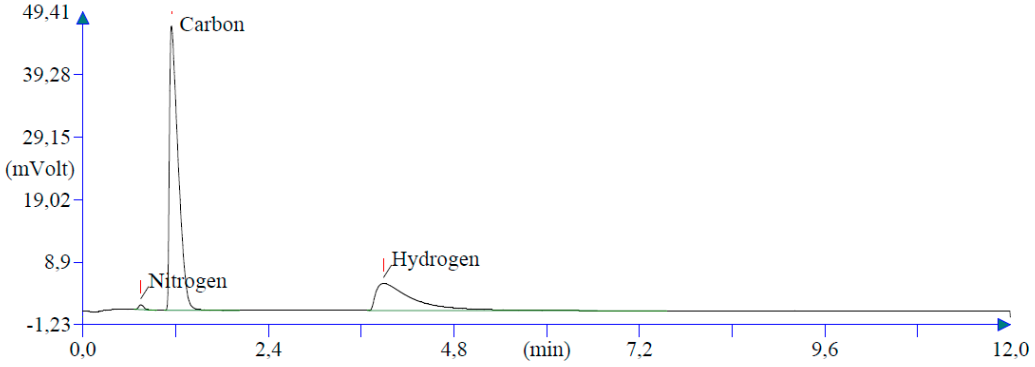

Element Name		Ret.Time	Area	BC	Area ratio	K f
Nitrogen	0.6121	45	36102	RS	99.982080	.236
Carbon	55.0871	69	3609553	RS	1.000000	.475
Hydrogen	6.2232	234	1454898	RS	2.480966	.151
Totals	74.6224		5100553			

c

Figure S25. Chemical characterization of **6**-poly(BuMA-*co*-EDMA) (**10**) (a) ATR-FT-IR spectra. (b) Raman spectra. (c) Elemental analysis.

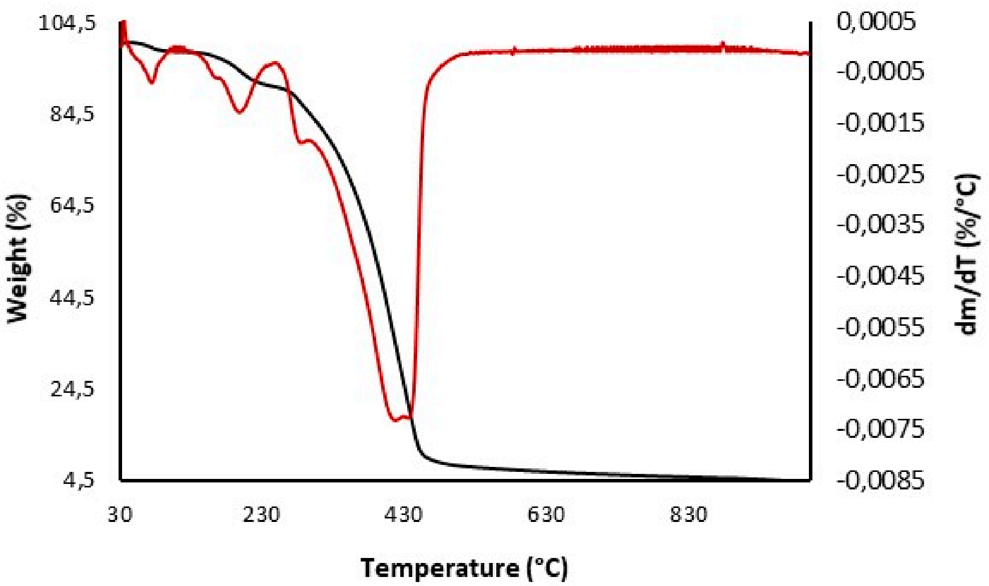

a

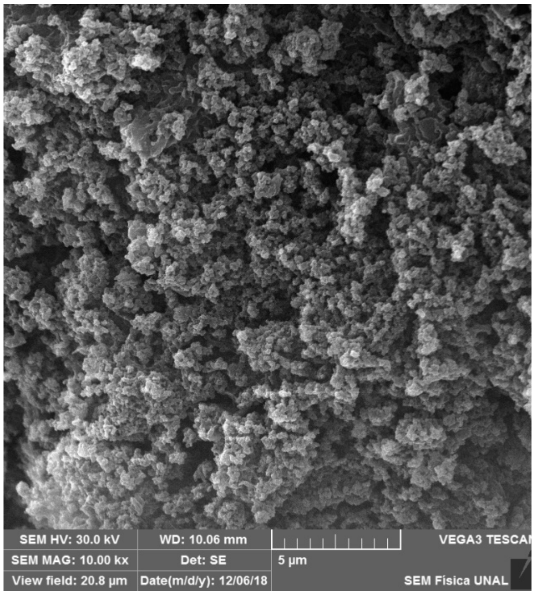

b

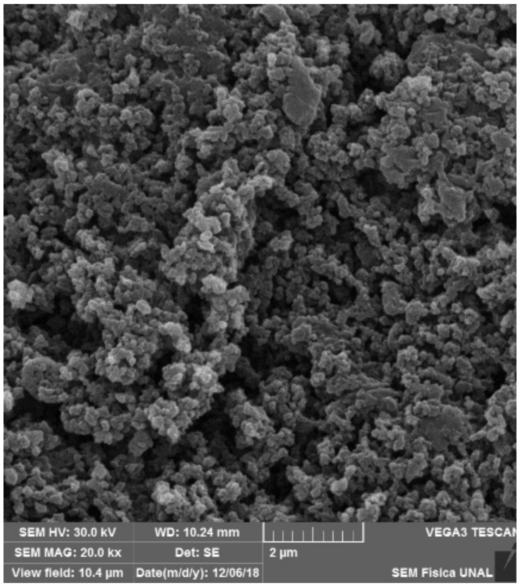

c

Figure S26. Thermal stability and morphological characterization of **6**-poly(BuMA-*co*-EDMA) (**10**). (a) Thermogram TGA (black) and curve dm/dT (red). Scanning electron micrograph at (b) 5 μm and (c) 2 μm.

Figure S27. Screening design matrix.

Exp.	Coded values				Natural values				Re (%)
	X_1	X_2	X_3	X_4	$m_{sorbent}$ (mg)	$t_{desorption}$ (min)	mM_{NH_4OAc} (mmol/L)	$V_{eluyent}$ (mL)	
1	-1	-1	-1	-1	20	10	0	5	52,8
2	1	-1	-1	-1	30	10	0	5	53,5
3	-1	1	-1	-1	20	50	0	5	63,5
4	1	1	-1	-1	30	50	0	5	65,1
5	-1	-1	1	-1	20	10	40	5	54,6
6	1	-1	1	-1	30	10	40	5	53,4
7	-1	1	1	-1	20	50	40	5	64,2
8	1	1	1	-1	30	50	40	5	66,3
9	-1	-1	-1	1	20	10	0	10	85,2
10	1	-1	-1	1	30	10	0	10	83,5
11	-1	1	-1	1	20	50	0	10	97,4
12	1	1	-1	1	30	50	0	10	97,8
13	-1	-1	1	1	20	10	40	10	82,5
14	1	-1	1	1	30	10	40	10	84,7
15	-1	1	1	1	20	50	40	10	96,5
16	1	1	1	1	30	50	40	10	98,6
17	0	0	0	0	25	30	20	7,5	92,5
18	0	0	0	0	25	30	20	7,5	93,2
19	0	0	0	0	25	30	20	7,5	90,8
20	0	0	0	0	25	30	20	7,5	91,4

Figure S28. Optimization design matrix.

Exp.	Coded values		Natural values		Re (%)
	X_1	X_2	$t_{desorption}$ (min)	$V_{eluyent}$ (mL)	
1	-1	-1	15	5	56,9
2	1	-1	45	5	81,6
3	-1	1	15	10	89,3
4	1	1	45	10	98,3
5	-1,41421	0	9	7,5	78,4
6	1,41421	0	51	7,5	98,5
7	0	-1,41421	30	4	75,8
8	0	1,41421	30	11	95,2
9	0	0	30	7,5	93,4
10	0	0	30	7,5	92,7

Figure S29. Standard calibration curves. In water (0.05% TFA) (red line) and on matrix (blue line).

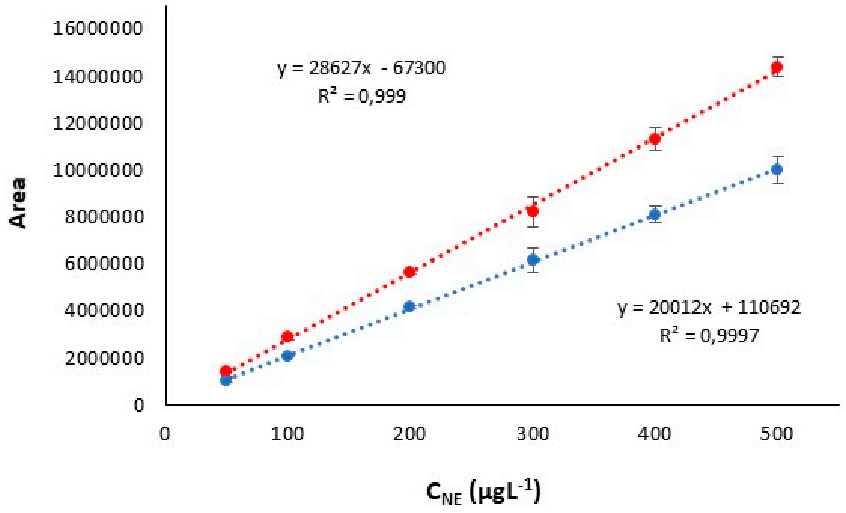

Figure S30. Calibration curve of fortified extracts.

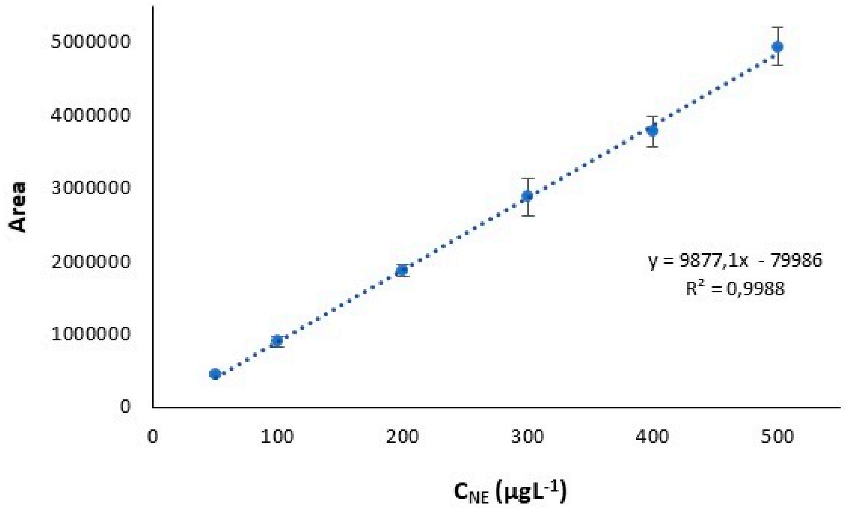

Supplement: Supplementary file 1 [file polymers-11-01428-s001.pdf]
